# AKT/mTOR signaling modulates resistance to endocrine therapy and CDK4/6 inhibition in metastatic breast cancers

**DOI:** 10.1038/s41698-023-00360-5

**Published:** 2023-02-16

**Authors:** Maysa M. Abu-Khalaf, K. Alex Hodge, Christos Hatzis, Elisa Baldelli, Emna El Gazzah, Frances Valdes, William M. Sikov, Monica M. Mita, Neelima Denduluri, Rita Murphy, Daniel Zelterman, Lance Liotta, Bryant Dunetz, Rick Dunetz, Emanuel F. Petricoin, Mariaelena Pierobon

**Affiliations:** 1 Sidney Kimmel Cancer Center at Thomas Jefferson University, Philadelphia, PA USA; 2 School of Systems Biology, Center for Applied Proteomics and Molecular Medicine, George Mason University, Fairfax, VA USA; 3 HiFiBiO Therapeutics, Cambridge, MA USA; 4 Sylvester Comprehensive Cancer Center (UM SCCC), University of Miami, Miami, FL USA; 5 Women and Infants Hospital of Rhode Island, Providence, RI USA; 6 Cedars-Sinai Medical Center, Los Angeles, CA USA; 7 Virginia Cancer Specialists, Fairfax, VA USA; 8 Yale University, New Haven, CT USA; 9 Side Out Foundation, Fairfax, VA USA

**Keywords:** Predictive markers, Breast cancer

## Abstract

Endocrine therapy (ET) in combination with CDK4/6 inhibition is routinely used as first-line treatment for HR+/HER2− metastatic breast cancer (MBC) patients. However, 30–40% of patients quickly develop disease progression. In this open-label multicenter clinical trial, we utilized a hypothesis-driven protein/phosphoprotein-based approach to identify predictive markers of response to ET plus CDK4/6 inhibition in pre-treatment tissue biopsies. Pathway-centered signaling profiles were generated from microdissected tumor epithelia and surrounding stroma/immune cells using the reverse phase protein microarray. Phosphorylation levels of the CDK4/6 downstream substrates Rb (S780) and FoxM1 (T600) were higher in patients with progressive disease (PD) compared to responders (*p* = 0.02). Systemic PI3K/AKT/mTOR activation in tumor epithelia and stroma/immune cells was detected in patients with PD. This activation was not explained by underpinning genomic alterations alone. As the number of FDA-approved targeted compounds increases, functional protein-based signaling analyses may become a critical component of response prediction and treatment selection for MBC patients.

## Introduction

Of the estimated 168,000 women currently living with metastatic breast cancer (MBC) in the United States, up to 70% have hormone receptor (HR) positive, HER2 negative (HER2-) tumors^1^. Several classes of compounds able to modulate estrogen activity or synthesis have been approved by the FDA over the past 40 years, and these drugs remain the treatment of choice for patients with HR-positive advanced breast cancers. While many patients initially respond to endocrine therapy (ET), intrinsic or acquired resistance often prevents long-term responses to these targeted compounds^2–5^.

Cell cycle dysregulation is a known hallmark of cancer and HR+ tumors rely on the interaction between estrogen and its receptor (ER) to promote proliferation^6^. Cyclin-dependent kinases (CDKs), a group of serine/threonine protein kinases, along with their specific cyclin-activating regulatory subunit are critical regulators of cell cycle progression^7–9^. In estrogen-driven breast tumors, CDK4, CDK6, and their substrate cyclin D1, a direct transcriptional target of ER signaling, enable G1 to S phase transition through post-translational modifications of downstream substrates like the hyperphosphorylation and inactivation of the retinoblastoma tumor suppressor protein Rb^5,7,10–12^. Based on preclinical and clinical studies indicating that signaling through the CDK4/6 pathway sustains cell growth and drives resistance to ET in breast cancer^13–17^, a number of compounds targeting CDK4/6 have been approved by the FDA in combination with ET as first and second-line treatments for patients with advanced disease. These combination treatments have nearly doubled progression-free survival in patients with advanced disease compared to single-agent ET^13,18–20^.

However, despite the improved clinical outcomes, 30–40% of patients with MBC show resistance to ET and CDK4/6 inhibition after a short time interval and experience progression-free survival (PFS) in <6 months^13–17^. With the growing number of effective targeted therapeutics beyond CDK4/6 inhibition available for patients with HR+/HER2− MBC^21,22^, finding biomarkers that can identify patients who are likely to rapidly progress on CDK4/6 inhibitors as front-line treatment remains an urgent need in oncology. Recent and past efforts to identify predictive biomarkers of response to CDK4/6 inhibition and ET have focused on genomic and transcriptional alterations associated with a lack of response to treatment. Due to their role as active regulators of CDK4/6 activity, amplification of *CCND1* or *CCNE1* as well as loss of *Rb1* and *p16 INK4A* (or *CDKN2A*) have been proposed as potential predictive markers of response to CDK4/6 inhibition in combination with ET^23–30^, but these markers have shown limited clinical utility^14,15,20,31–33^.

We hypothesized that the functional activation state of signaling molecules driving biochemical cascades involved in CDK4/6 signaling as well as survival and metastatic progression hold predictive value for a patient’s response to treatment to CDK4/6 inhibition in combination with ET in MBC patients. To test our hypothesis, we conducted an open-label, multicenter clinical trial and mapped functional activation and signaling architecture of pretreatment tumor tissue biopsies collected from HR+/HER2− MBC patients receiving first-line treatment with a CDK4/6 inhibitor plus ET. Our analysis suggests that expression/activation of CDK4/6 substrates in tumor cells and genomic-independent activation of the AKT/mTOR pro-survival signaling pathway in tumor and surrounding stroma/immune cells may predict response to CDK4/6 inhibition in combination with ET in MBC.

## Results

### Trial enrollment and patients’ clinical characteristics

Between April 2017 and July 2020, a total of 41 newly diagnosed metastatic breast cancer patients were screened for study eligibility (Supplementary Table 1). While the study was originally designed to enroll 100 patients to achieve a statistical power of 80% for the qualifying biomarker analysis, due to slow accrual, the study was prematurely closed. Of the 41 patients screened, 27 met study eligibility criteria and 20 of 27 patients were evaluable for response assessment as outlined per study protocol and were included in this biomarker analysis. Of the 7 patients that were not included in the final analysis, two had insufficient biological material, two withdrew from the study, two became eligible for surgery and one had a change of treatment plan. All 20 evaluable patients were female and the median age was 67.5 (range between 36-79). Six patients (30%) were African-American and 14 (70%) were White (Table 1); three of the evaluable patients were Hispanic. Thirteen patients (65%) were diagnosed with a de novo stage IV breast cancer and had received no prior treatment. Of the 20 samples included in the analysis, only one sample was classified as lobular (Table 1). Lobular, sebaceous, and neuroendocrine features were identified in one invasive ductal carcinoma, respectively. Thus, the molecular profiles emerging from our analysis are most likely independent of the underlying histological characteristics of the lesions analyzed. Patients received a median number of 12 cycles (range 3 to 12 cycles) of treatment while in the study. The most frequently used regimen was letrozole plus palbociclib was given to 15 (75%) patients followed by fulvestrant plus palbociclib for 2 (10%) patients. The remaining three patients were treated with anastrozole plus palbociclib, letrozole plus abemaciclib, and letrozole plus ribociclib, respectively (Table 1).

**Table 1:** Demographic characteristics, treatment regimens, and response class for 20 evaluable patients included in the study.

Patient ID	Response Class	Age	Sex	Race	Target lesion(s)	Site of biopsy	Histology	Bone mets	Endocrine therapy	Cdk4/6 inhibitor (i)	Months on trial	No. cycles
03-01-001	PD	58	F	White	LN	LN	IDC	Y	Fulvestrant	Palbociclib	6.3	6
03-02-006	PD	48	F	White	Liver, Breast	Liver	Carcinoma NOS	Y	Letrozole	Ribociclib	3.1	3
03-01-010	PD	70	F	Black	Lung	LN	Adenoca NOS	Y	Letrozole	Palbociclib	7.3	6
03-01-032	PD	68	F	White	Lung	Pleura cavity	Carcinoma NOS	N	Letrozole	Palbociclib	5.7	6
03-04-013*	SD	62	F	White	Breast	Breast	IDC	Y	Letrozole	Palbociclib	13.5	12
03-08-015*	SD	63	F	White	Pectoralis	LN	Adenoca NOS	N	Letrozole	Palbociclib	11	12
03-08-017*	SD	59	F	White	LN, Breast, Bone, Lung	Breast	IDC	Y	Letrozole	Palbociclib	11	12
03-08-021	SD	68	F	White	Lung, Breast	Breast	IDC	Y	Letrozole	Palbociclib	10.9	12
03-01-026*	SD	79	F	Black	LN, Bone, Liver	Breast	IDC; lobular features	N	Fulvestrant	Palbociclib	10.9	12
03-01-029*	SD	67	F	Black	N/A	Breast	IDC; lobular features	Y	Letrozole	Palbociclib	13.4	12
03-01-008*	PR	36	F	White	LN, Adrenal	LN	IDC; NE differentiation	Y	Letrozole	Palbociclib	12.6	12
03-01-009*	PR	54	F	Black	Breast	LN	IDC	Y	Letrozole	Palbociclib	13.7	12
03-04-022*	PR	76	F	White	Breast, LN	LN	Carcinoma NOS	N	Letrozole	Palbociclib	11.2	12
03-08-025*	PR	72	F	White	Bone, Liver, Lingular	Breast	IDC	Y	Letrozole	Palbociclib	10.9	12
03-01-028*	PR	75	F	White	LN, Breast	Breast	IDC	Y	Letrozole	Palbociclib	12.9	12
03-01-039*	PR	70	F	Black	Breast, LN, Lung	Breast	Carcinoma; sebaceous features	Y	Letrozole	Palbociclib	11	12
03-04-040*	PR	68	F	White	Breast, LN,	Breast	IDC; micropapillary features	N	Letrozole	Abemaciclib	11.2	6
03-10-033*	CR	55	F	White	LN	Breast	Invasive lobular carcinoma	Y	Anastrozole	Palbociclib	11.3	12
03-08-036	CR	74	F	White	Liver	Liver	Adenoca NOS	Y	Letrozole	Palbociclib	9.7	12
03-04-038	CR	48	F	Black	Breast, LN	Breast	IDC	N	Letrozole	Palbociclib	11.4	7

*IDC* invasive ductal carcinoma, *LN* lymph node, *Y* present, *N* absent, *NOS* not otherwise specified.

*De novo diagnosis.

During the 12-month follow-up from treatment initiation, 16 (80%) of the 20 patients responded to treatment while 4 (20%) patients developed progressive disease (PD). Median age, presence of lung, liver, and bone metastases, and type of combination treatment received did not differ between responders and patients with PD (*p* > 0.05). Amongst responders, 6 (37%) had stable disease (SD), 7 (44%) had a partial response (PR), and 3 (19%) had a complete response (CR) for at least 1 year from treatment initiation. A summary of study-related and unrelated adverse events (AEs) can be found in Supplementary Table 3.

Of the seven patients with recurrent diseases, five had previously received adjuvant treatment; three patients were treated with chemotherapy and ET, and two patients with adjuvant ET only. Four patients (57%) with recurrent disease developed PD and three (43%) benefitted from treatment for the entire duration of the trial. Amongst responders with recurrent disease, two achieved a CR and one had SD. Although none of the patients with a de novo stage IV breast cancer developed PD within the 12-month window, suggesting a potential association between response and previous treatments, only one of the de novo patients achieved CR compared to two patients with recurrent disease, one of which had received adjuvant ET.

### Lack of response to ET and CDK4/6 inhibitors in MBCs is associated with the expression and phosphorylation of CDK4/6 downstream substrates

**Fig. 1:**
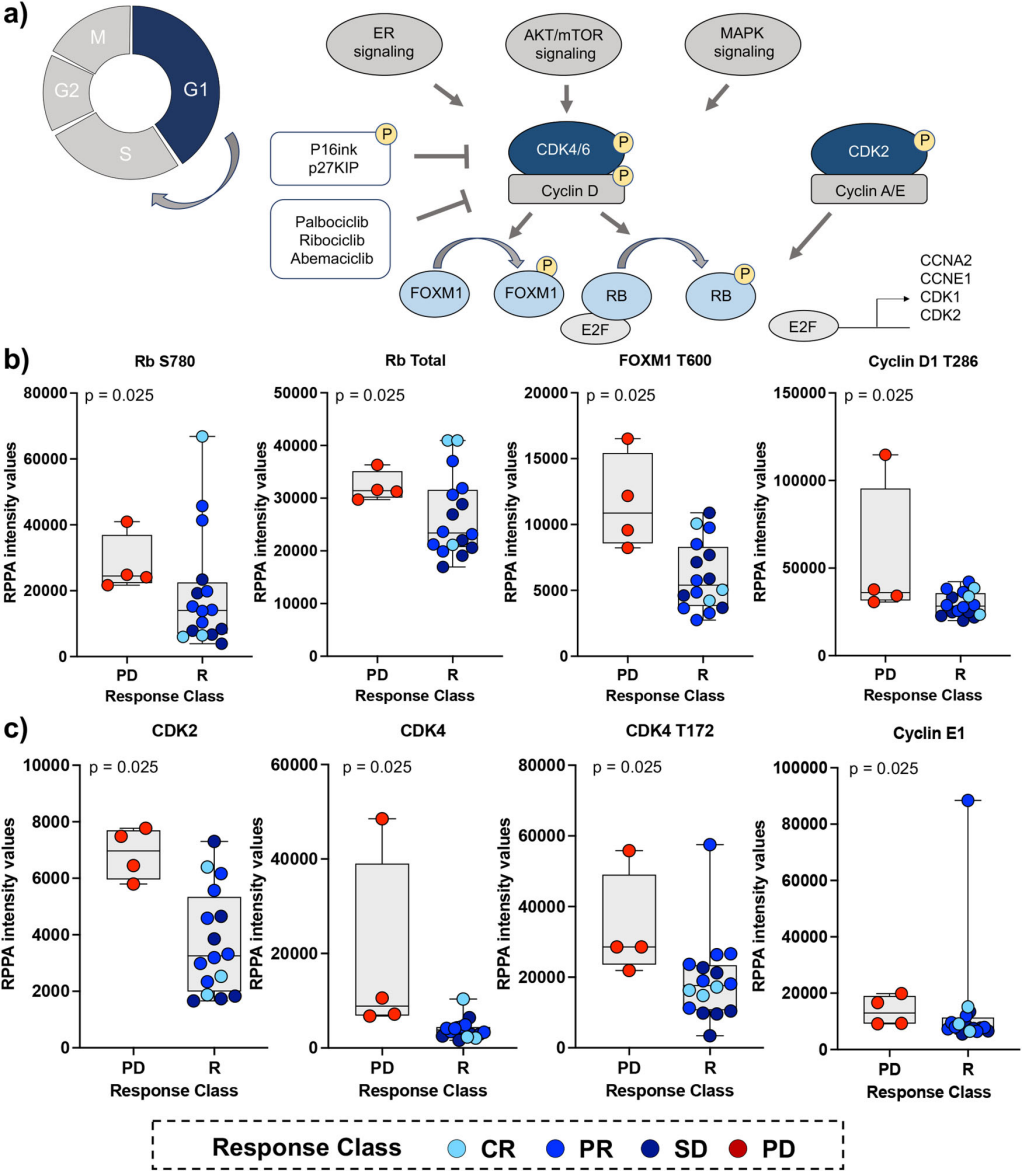
Expression and phosphorylation levels of CDK4/6 downstream substrates and regulators correlate with response to first-line treatment with ET plus CDK4/6 inhibition in MBC patients. Diagram showing functional interactions between CDK4/6 and its downstream substrates (**a**). Box plots with median (center line) and maximum and minimum values (whiskers) show RPPA-based expression and activation levels of CDKs, cyclins, and their downstream substrates. RPPA-based expression and/or phosphorylation of key functional residues of three qualifying biomarkers was significantly higher in patients with progressive disease (PD) compared to responders (R) (**b**). A statistically significant increase in the expression and activation of signaling molecules involved in Rb regulation was also detected in patients with progressive disease compared to responders (**c**); *p* values for two-sided chi-square tests for dichotomized levels of measured biomarkers (above/below the population median) are shown. Samples are color-coded based on patient outcomes.

The main objective of this trial were to assess whether baseline expression and phosphorylation levels of eight known CDK4/6 substrates and regulators are associated with response to ET in combination with an FDA-approved CDK4/6 inhibitor. A priori selected qualifying biomarkers included phosphorylated Rb (S780), FoxM1 (T600), p27 Kip1 (T187), cyclin D1 (T289), and unmodified Rb, cyclin D1, p27 Kip1, and p16 INK4a (Fig. 1a). Given that Rb is a major direct downstream substrate of CDK4/6 activity, differences in phosphorylation of Rb at the residue S780 in responders and non-responders were explored as the primary objective of the study. RPPA data were captured on a continuous scale. However, expression/phosphorylation of the qualifying biomarkers was dichotomized as high/low based on the population median to increase the power of the analysis due to the small size of the study.

Phosphorylation of Rb at the S780 residue, and its consequent inactivation, was significantly different between responders (CR, PR, SD) and non-responders (PD) (*p* = 0.025) (Fig. 1b). Patients with early disease progression were more likely to exhibit high levels of Rb inactivation compared with responders. Similar results were also detected for unmodified Rb and post-translationally modified FoxM1 (T600) and cyclin D1 (T289) (*p* = 0.025 for all biomarkers) (Fig. 1b). Taken together, these data suggest that increased phosphorylation of direct substrates of CDK4/6 activity may be associated with lack of response to ET in combination with a CDK4/6 inhibitor.

### Response to ET in combination with a CDK4/6 inhibitor in MBCs is associated with baseline kinase-driven signal transduction events

**Fig. 2:**
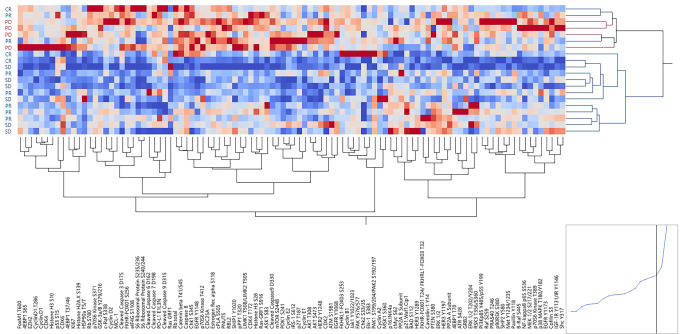
Expression and activation levels of qualifying and exploratory biomarkers in the tumor epithelia of responders and non-responders. Unsupervised hierarchical clustering analysis using Ward’s method displaying unmodified and post-translationally modified kinases and their substrates in the tumor epithelia of MBC patients measured by RPPA. RPPA relative intensity values are displayed on a scale ranging from red to blue, where red indicates high levels and blue indicates low levels of expression/activation. On the x-axis are listed proteins measured by RPPA; on the y-axis patients’ outcomes are displayed; samples were color-coded based on response rates where blue indicates responders (CR, PR, and SD) and red non-responders (PD).

We then conducted an exploratory analysis to capture kinase-driven, pathway-centered mechanisms of resistance to CDK4/6 inhibition plus ET in our cohort of patients. Unmodified epitopes and post-translationally modified residues of 126 proteins were measured by RPPA. Unsupervised hierarchical clustering analysis was first used to broadly capture signaling events associated with response to treatment. While clear clustering patterns were not detected based on the biopsy site (Supplementary Fig. 2), at large, tumors from patients with PD were contained within the same cluster and showed overall higher levels of expression and activation across signaling molecules (Fig. 2).

To capture changes in signal transduction events associated with response to treatment, the proportion of patients with high and low expression of each biomarker was compared based on patients’ outcomes. Of the 126 proteins quantified by RPPA, 61 emerged as statistically significant between responders and non-responders (Table 2).

**Table 2:**
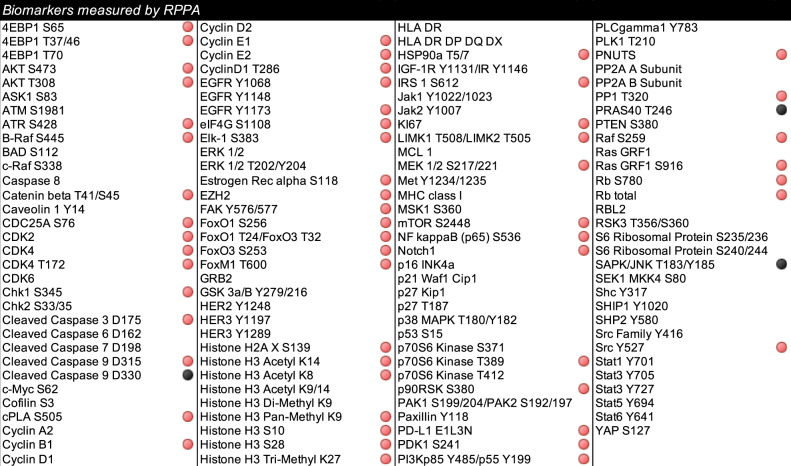
List of proteins that reached statistical significance when responders were compared to patients with progressive disease.

Samples were cross-classified as above/below median values. The chi-square test was used to compare biomarkers’ distributions in responders and non-responders. Red circles indicate proteins that in the comparison reached a *p* value <0.05 and black circles indicate proteins that in the comparison reached a *p* value <0.01.

Expression and activation levels of proteins known to be involved in the regulation of Rb were increased in patients who progressed compared to responders (*p* = 0.025), including CDK2, CDK4, CDC25A, cyclin E1, and cyclin E2 (Fig. 1c). This data suggests that deactivation of Rb and cell cycle progression in patients that develop resistance to treatment may be driven by CDK4/6-dependent and -independent mechanisms. As expected, reduced Rb activity was associated with increased proliferation rates shown by greater expression of Ki67 in patients who developed PD within the first 12 months of treatment (Supplementary Fig. 1A). Post-translational modifications, including phosphorylation, acetylation, and methylation, of Histone H3 were also significantly different between responders and non-responders, suggesting that chromatin accessibility and chromosome condensation patterns differ based on patients’ outcomes (Supplementary Fig. 1A and Table 2). Lastly, the proportion of patients with high activation levels of proliferative signaling molecules including EGFR (Y1068 and Y1173) and its downstream substrates B-Raf (S445) and MEK 1/2 (S217/221) were also greater in patients with PD compared to responders (p = 0.025, for all molecules) (Table 2). Phosphorylation of the S118 residue on Estrogen Receptor alpha, a residue that is a known target of many kinases including MAPK activity^34^, was also greater in patients with PD compared to responders (Supplementary Fig. 1B).

### Baseline global activation of the PI3K/AKT/mTOR signaling axis in the tumor epithelia of MBCs is associated with response to CDK4/6 inhibition in combination with ET

**Fig. 3:**
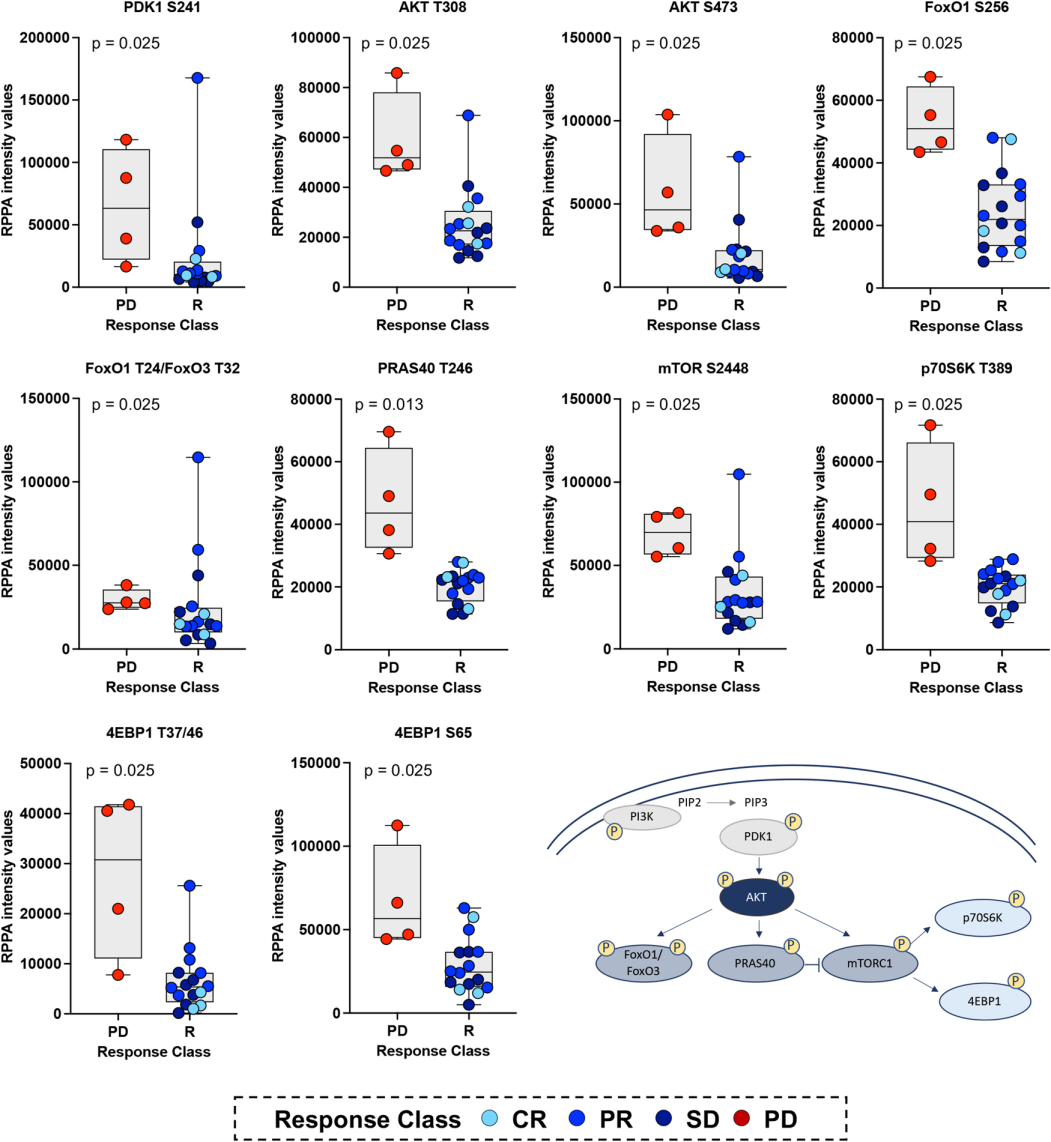
Global activation of the PI3K/AKT/mTOR signaling pathway in patients with progressive disease compared to responders. Box plots with median (center line) and maximum and minimum values (whiskers) show a statistically significant increase in the activation levels of many members of the PI3K/AKT/mTOR signaling pathway in the tumor epithelium of patients with progressive disease compared to responders; *p* values for two-sided chi-square tests for dichotomized levels of measured biomarkers (above/below the population median) are shown.

Along with the deregulation of the cell cycle progression, tumors collected from patients with PD were also characterized by a global activation of the PI3K/AKT/mTOR signaling cascade. Of the 61 proteins that emerged as statistically significant, 15 were members of the PI3K/AKT/mTOR signaling axis (Fig. 3). Specifically, patients with PD had increased activation of AKT measured by the phosphorylation levels of the S473 and T308 residues (*p* = 0.025) along with its regulator PDK1 (S241) (*p* = 0.025) (Fig. 3). Increased levels of phosphorylation were also detected for the AKT substrates FoxO1 (S256), FoxO1 (T24)/FoxO3 (T32), FoxO3 (S253), mTOR (S2448) (*p* = 0.025), and the mTOR regulator PRAS40 (T246) (*p* = 0.013) (Fig. 3). Finally, activation of the mTORC1 substrates p70S6K (T389) and 4EBP1 (S65) and (T37/46) (*p* = 0.025) were also increased in samples collected from patients with PD.

**Fig. 4:**
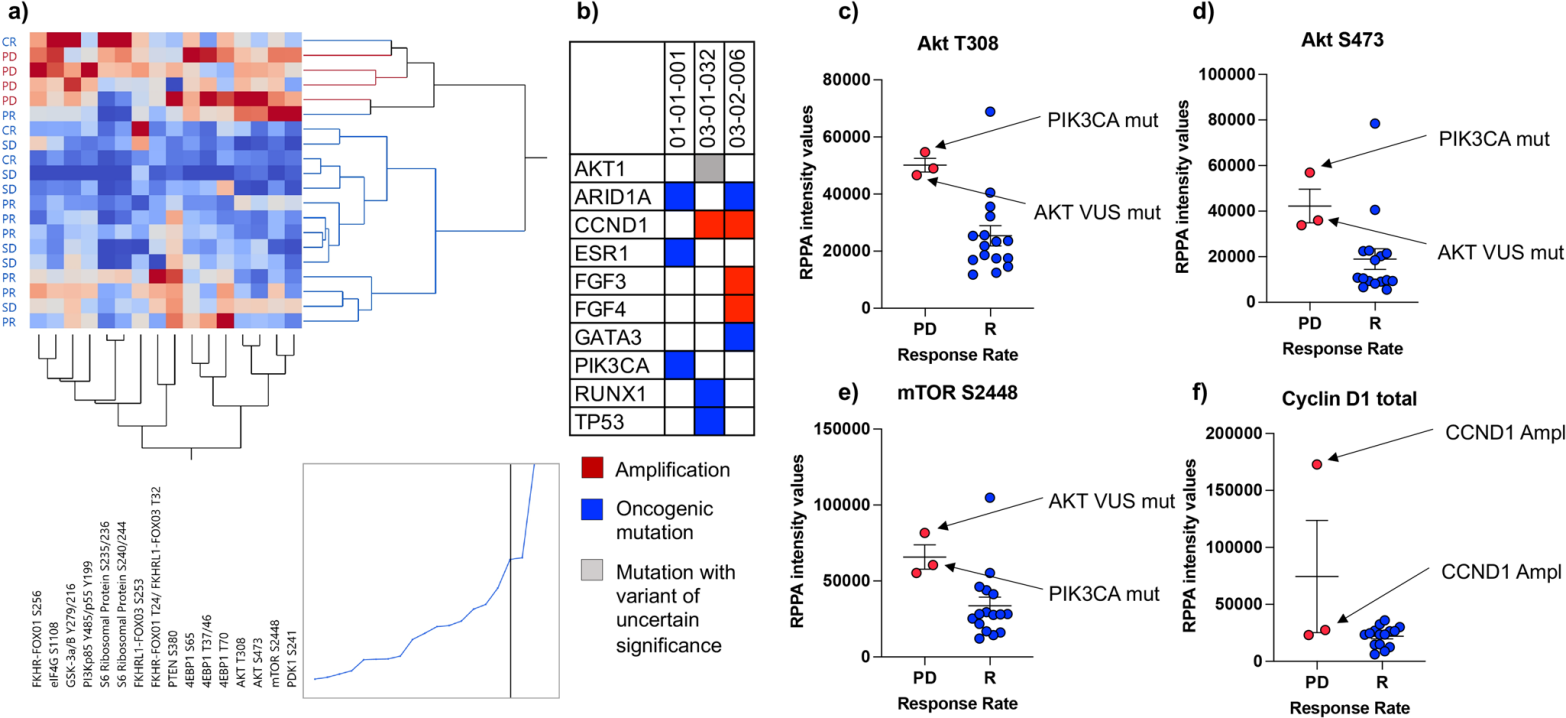
Correlation between oncogenic genomic alterations and protein expression/activation in patients with progressive disease. Unsupervised hierarchical clustering analysis using Ward’s method of members of the PI3K/AKT/mTOR signaling pathway shows increased activity and clustering of tumor epithelia collected from patients that developed disease progression (**a**). NGS-based analysis of the recurrent lesions at disease progression for three of the four patients with progressive disease identified an oncogenic mutation of *PIK3CA* in one patient and amplification of the *CCND1* gene in the remaining two patients (**b**). Increased activation levels of AKT and mTOR detected in patients with progressive disease did not correlate with the underlying genotype (**c**–**e**). Similarly, the expression of Cyclin D1 did not directly correlate with *CCND1* amplification (**f**).

As expected, when unsupervised hierarchical clustering analysis was applied to this data set, tumors collected from patients with PD clustered together (Fig. 4a). When multiple phosphorylation sites of the same protein were measured (e.g., AKT, 4EBP1, S6 ribosomal protein), these analytes also clustered together suggesting distinct patterns of activation of these signaling molecules across samples.

To further assess the role of the PI3K/AKT/mTOR signaling axis in modulating response to treatment in our cohort of patients, we next assessed whether activation of this signaling cascade could be explained by underlying genomic oncogenic alterations of different members of this signaling pathway. Based on the study protocol, at the time of recurrence, patients were eligible for an additional biopsy. Tissue samples collected at the time of recurrence were used to generate NGS and proteomic/phosphoproteomic-based molecular profiles to inform future treatment selections. Of the four patients with PD, three opted for the optional biopsy, and tissue specimens were used to generate a panel-based NGS profile of drug targets’ predictors. All tumors were microsatellite stable and the tumor mutational burden was intermediate for two patients and intermediate/low for the third patient. A full list of the genomic alterations identified is provided in Fig. 4b. Although deactivation of Rb was higher in patients with PD compared to responders in our data set (Fig. 1b), none of the patients with recurrent disease harbored oncogenic alterations of the Rb gene, suggesting that post-translational modification drives Rb activity in these tumors regardless of the underlying genotype.

Of interest, while activation of the PI3K/AKT/mTOR signaling was increased and relatively comparable across all three patients with PD compared to responders, only one of the three patients harbored an oncogenic mutation of the *PIK3CA* gene (Fig. 4c–f). A second patient presented with a variant of uncertain significance (VUS) of the *AKT1* gene; however, this variant was not considered a drug target predictor. A lack of concordance was also observed when the amplification of the *CCND1* gene, which was present in two of the three patients, was correlated to cyclin D1 expression measured by RPPA (Fig. 4f). Taken together, our data suggests that global baseline activation of the PI3K/AKT/mTOR signaling axis may be associated with short-term or lack of response to ET in combination with CDK4/6 inhibitors. However, the activation of this signaling cascade in patients that developed PD is driven by genomic-dependent and independent mechanisms. Thus, its relevance as a predictor of response to treatment may be underestimated when genomic analyses are used as the sole source of molecular information.

### Activation of the PI3K/AKT/mTOR signaling axis in patients that develop disease progression expands beyond the malignant cells into the surrounding stroma

**Fig. 5:**
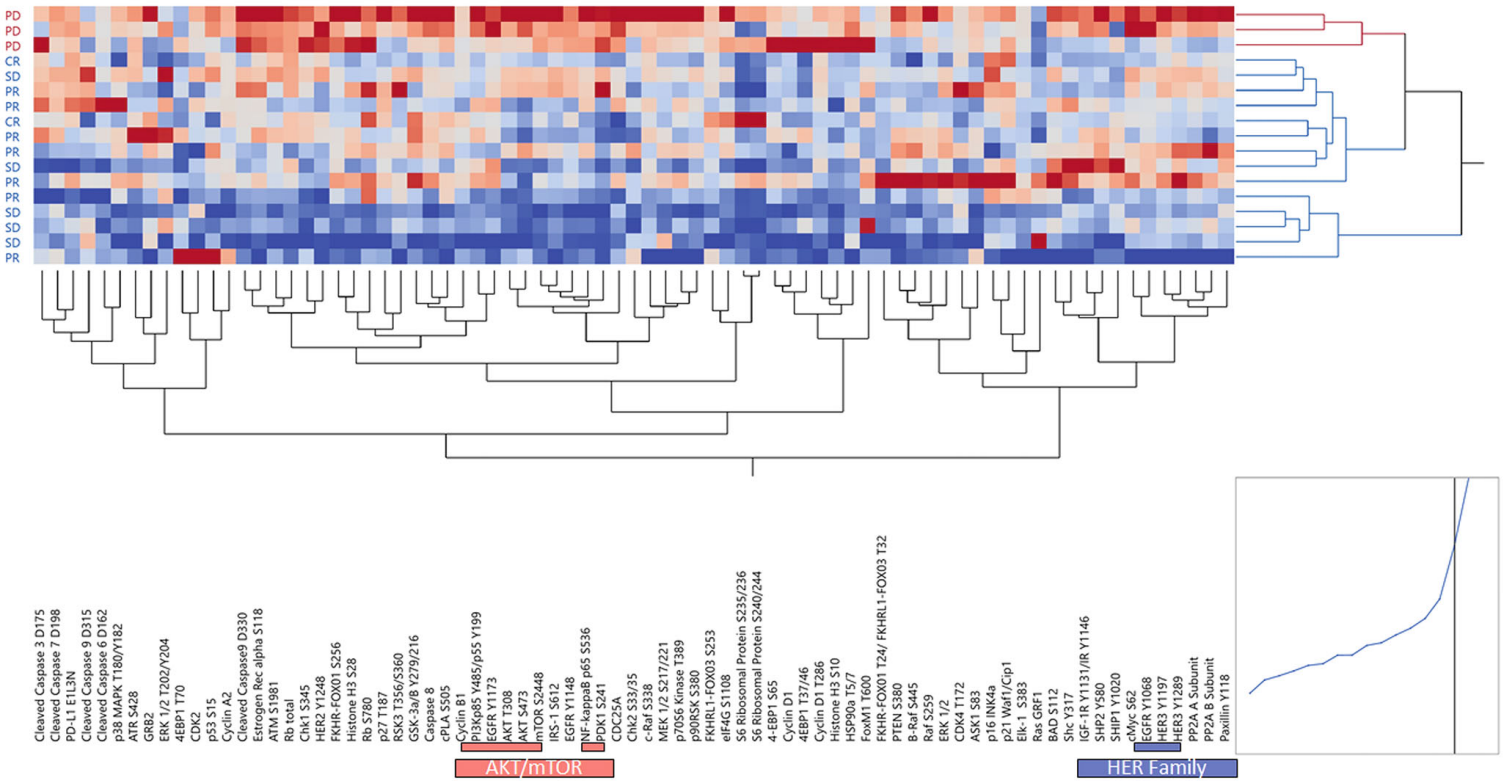
Expression and activation levels of qualifying and exploratory biomarkers in the stroma of responders and non-responders. Unsupervised hierarchical clustering analysis using Ward’s method displays kinases and their substrates in the microdissected stroma surrounding the tumor cells collected from 17 of the 20 evaluable patients. RPPA relative intensity values are displayed on a scale ranging from red to blue, where red indicates high levels and blue indicates low levels of expression/activation. On the x-axis are listed proteins measured by RPPA; on the y-axis patients’ outcomes are displayed; samples were color-coded based on response rates where blue indicates responders (CR, PR, and SD) and red non-responders (PD). Members of the PI3K/AKT/mTOR signaling pathway and HER family co-clustering together are highlighted on the x-axis in red and blue, respectively.

**Fig. 6:**
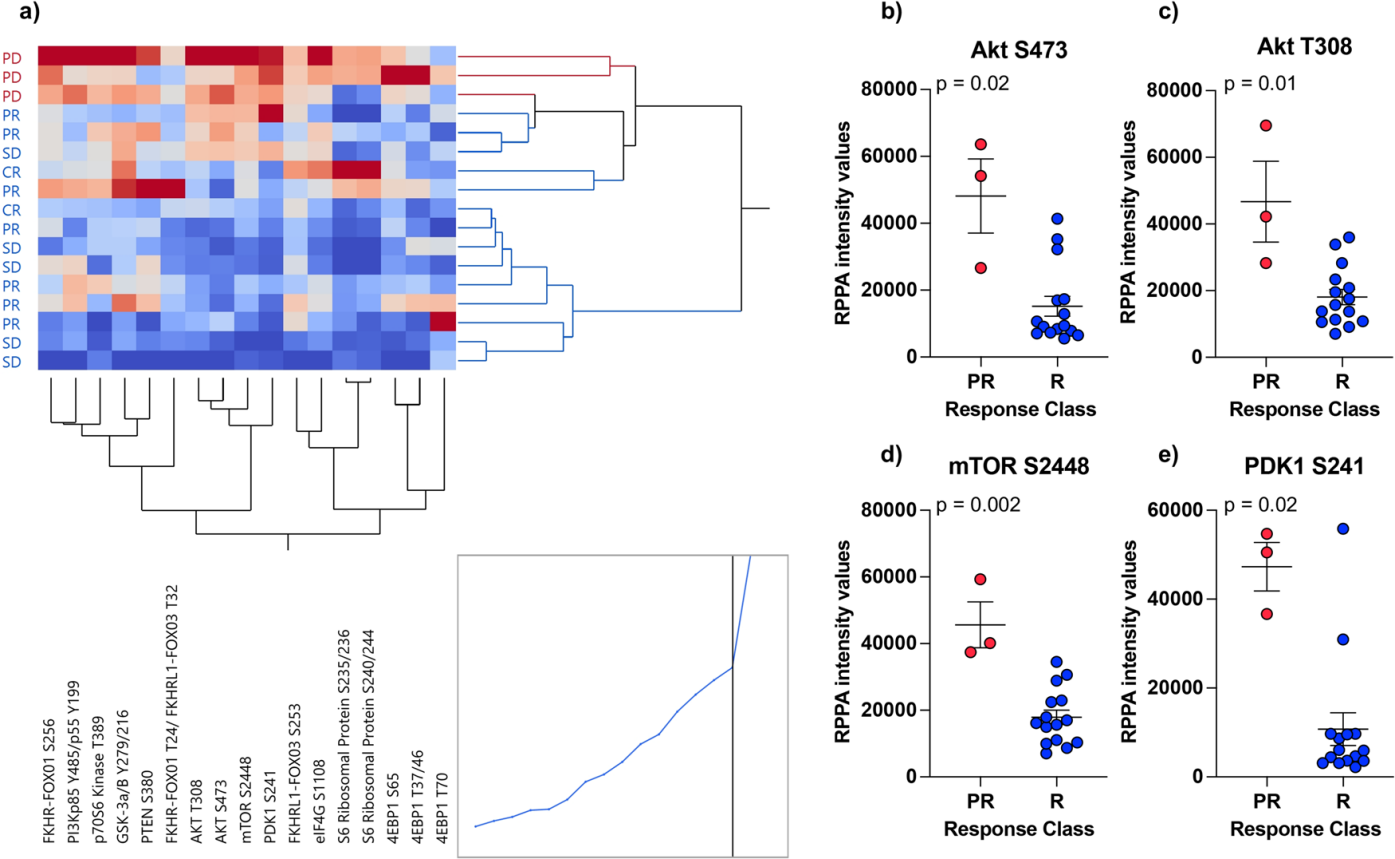
Activation of the PI3K/AKT/mTOR signaling pathway in the stroma surrounding the tumor of patients with progressive disease compared to responders. Unsupervised hierarchical clustering analysis using Ward’s method shows an overall increase in PI3K/AKT/mTOR signaling activity in the stroma of patients that developed progressive disease (**a**). Scatter plots with mean and standard error of the mean showing activation levels of key members of the signaling pathway (**b**–**e**).

For 17 of the 20 (85%) eligible patients, matched stroma cells surrounding the tumor epithelia were also collected using LCM and processed as separate tissue compartments. For the remaining three samples, the stroma component was poorly represented in the biospecimens and was insufficient for molecular analysis. Unsupervised hierarchical clustering analysis of the stroma compartment showed a clear separation between samples collected from responders versus those collected from patients that developed PD within the first 12 months after study enrollment (Fig. 5). Because many members of the PI3K/AKT/mTOR signaling axis were contained within the same cluster and showed overall increased activation in patients with disease progression, we performed a focused analysis of this signaling axis. As seen for the epithelial component, patients with PD had an overall increased activation of AKT, mTOR, and their downstream substrates (Fig. 6a). When the activation of selected proteins was compared between responders and patients with PD using the non-parametric Mann–Whitney test, all comparisons reached statistical significance (Fig. 6b/e). Taken together, our data suggest that the global activation of the PI3K/AKT/mTOR signaling axis in tumors with short-term/lack of response to CDK4/6 inhibition in combination with ET extends beyond the tumor compartment into the surrounding tumor microenvironment. Thus, the activation of this signaling axis, and consequent poor response to treatment, may be highly driven by genomic-independent events.

## Discussion

To our knowledge, this is the first study assessing the protein signaling architecture of tumor epithelia and stroma cells in baseline clinical biospecimens as potential predictors of response to CDK4/6 inhibition in combination with ET in MBC patients. Given that Rb is a main downstream target of CDK4/6 activity, the primary objective of the study was to demonstrate the correlation between baseline Rb phosphorylation levels and one-year PFS in MBC patients. Going into our analysis, one plausible hypothesis was that tumors with high levels of Rb phosphorylation prior to treatment might be more sensitive to CDK4/6 inhibition; demonstrating the opposite suggests that, in these cancers, CDK4/6 inhibitors do not sufficiently block Rb phosphorylation to promote binding of the E2F transcription factor and impede cell cycle progression.

Based on the landmark PALOMA-3 study, loss of *Rb1* is present in ≤5% of patients who progress on palbociclib plus fulvestrant^35^, limiting its clinical utility as a predictive marker of response. In our cohort of patients, hyperphosphorylation of Rb at the S780 residue^36^, and its consequent inactivation^36^, was elevated (above the population median) in all tumors collected from patients that developed PD. Of interest, patients with PD had higher expression and activation of CDK4 as well as expression of CDK2 and its binding partner cyclin E1, two cell cycle regulators that can directly modulate Rb activity and bypass CDK4/6 inhibition^35,37^. Similar trends were also recently reported by Palafox et al. on a small cohort of advanced breast cancer patients^38^. In line with our findings, samples collected from patients that experienced resistance to treatment had greater levels of cyclin E, phosphorylated Rb, and p16, although we did not observe an association of p16 protein expression with the outcome in our analysis^38^. Thus, genomic-independent mechanisms, potentially through the activation of distinct CDK/cyclin complexes, modulate Rb activity and a tumor’s susceptibility to CDK4/6 inhibition^35,38–41^. To fully assess Rb activity in individual patients and predict response to anti-CDK4/6 treatment in combination with ET, genomic screening should be combined with quantification of Rb expression and inactivation at the protein level^42^.

Along with the inactivation of Rb, our data also suggest that in MBC patients, functional proteome pathway-centered signatures have distinct patterns of activation in responders and non-responders. Our data clearly showed functional upregulation of the PI3K/AKT/mTOR signaling axis in patients with PD with independent members of the pathway associating with clinical response. Activation of the PI3K/AKT/mTOR signaling network is a well-known mechanism of resistance to ET and of a number of chemotherapies. Activation of this pro-survival signaling axis has previously been reported as a resistance path to CDK4/6 inhibition in tumors with somatic mutations of *PIK3CA*, *AKT1*, or *PTEN*^43–47^. However, based on our observations, the systemic activation of the PI3K/AKT/mTOR pathway in tumors from patients with PD was not entirely attributable to the underlying genomic profile. These findings support previous data by our group and others demonstrating that the functional signaling activation of the PI3K/AKT/mTOR pathway in breast cancers, including in metastatic lesions, cannot be fully explained by the underlying genomic profile of the tumor^48,49^. Increased PI3K/AKT/mTOR signaling activity in stroma/immune cells surrounding the tumor cells further validates the role of genomic-independent mechanisms within the tumor microenvironment as key regulators of the PI3K/AKT/mTOR signaling cascade.

While our data provide critical insights on the role of the PI3K/AKT/mTOR signaling pathway in modulating response to treatment, a limitation of our work worth addressing is the relatively small sample size. Because of the relatively small cohort of patients and the limited number of patients with PD disease, the molecular data collected as part of this analysis show some degree of overlap between patients with PD and those that benefited from treatment (Fig. 3). However, even if our findings are based on a relatively small number of observations and need to be validated in a larger cohort of samples, our data expand on preclinical work recently published by Palafox et al. which showed that inhibition of PI3K sensitizes ribociclib-resistant ER-positive breast cancer PDX models to anti-CDK4/6 treatment. Of clinical relevance and in support of our observations, therapeutic responses to this combination treatment were independent of underlying oncogenic alterations of *PIK3CA* and *ESR1* or phospho-Rb expression^38^.

**Fig. 7:**
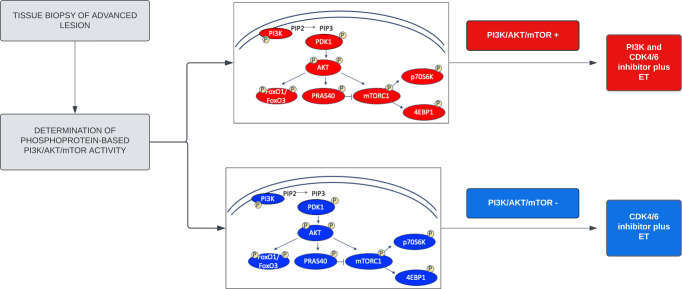
Decision-making workflow for selecting treatment for newly diagnosed ER+/HER2- metastatic breast cancer patients. Phosphoproteomic-based quantification of PI3K/AKT/mTOR activity in tumor samples using the LCM/RPPA workflow allows to identify patients with high pathway activation that may benefit from the addition of a PI3K inhibitor to sensitize resistant tumors to ET in combination with CDK4/6 inhibition.

In support of the unique role of phosphoprotein-based biosignatures as predictors of response to treatments targeting different members of the PI3K/AKT/mTOR signaling axis, a biomarker analysis conducted as part of the I-SPY 2 trial assessing response to the AKT inhibitor MK2206 in the neo-adjuvant setting, found a significant association between subtype-specific response rates and phosphorylation levels of AKT and its substrates^50^. Similarly, the FAIRLANE trial, where the AKT inhibitor ipatasertib was given in combination with paclitaxel as a neo-adjuvant treatment for triple-negative breast cancer patients, demonstrated that activation of the PI3K/AKT/mTOR signaling axis at the protein level was not fully predicted by genomic information alone. Of clinical significance, phosphorylation levels of AKT were associated with clinical response to ipatasertib independently of the underlying *PIK3CA*/*AKT* genomic profile or PTEN status^49^. If validated in larger cohorts of patients, these data suggests that to identify predestined resistance and stratify patients to therapy^38^, future treatment management plans for MBC patients should include upfront functional quantification of PI3K/AKT/mTOR signaling activity (Fig. 7).

However, capturing functional activation of protein drug targets and their biochemically linked downstream substrates in clinical samples using FDA-approved assays, like immunohistochemistry (IHC), remains challenging from a technical perspective. Kinase drug targets, like members of the PI3K/AKT/mTOR signaling axis, are extremely low abundance proteins often expressed at levels that are below the limit of detection of many proteomic assays^51^. In addition, the semiquantitative read-out of IHC and the need for antibody-specific antigen retrieval methods for the detection of post-translationally modified residues often prevent accurate quantifications of these drug targets in human biospecimens^51–53^. The LCM-RPPA workflow utilized was specifically developed to overcome some of these technical barriers and is currently offered as a standardized CLIA/LDT assay that can be used to identify druggable oncogenic events in tissue samples^50,54–57^. In this calibrated format of the RPPA assay, samples are immobilized along with internal controls for QA/QC and reference standard calibration curves established using a combination of commercially available cell lines containing different amounts of the analytes of interest, as previously described^48^. This calibrated format of the assay allows for transformation of RPPA-based absolute intensity values into interpolated relative intensity units of the standard curves and can be compared across independent experiments as shown in Supplementary Fig. 3. Thus, if our findings are validated in independent cohorts of patients, the standardized format of the assay can be easily used in future investigations to assess phospho-RB and PI3K/AKT/mTOR protein signaling activity for response prediction and first-line treatment selection in MBC patients^48,58–61^. If the global activation of the PI3K/AKT/mTOR signaling axis in the tumor epithelium and stroma compartment is validated in independent cohorts of samples, this assay may provide clinically useful information even from whole tissue samples without requiring additional cellular enrichment steps. Cut-points established on this platform can either be used on the standardized format of LCM-RPPA assay itself or be cross-walked to quantitative IHC formats as previously described^62^.

As the number of FDA-approved therapies for MBC with HR+/HER2− disease expands from the ER and CDK4/6 directed landscape to include targeting TROP2 and HER2^21,22^, a molecularly rationalized therapeutic selection process that captures activation levels of drug targets and substrates will become increasingly important given the disparate mechanisms of action of these therapies.

## Methods

### Trial design and patient eligibility criteria

This prospective open-label, non-randomized multicenter study (Clinical trial.gov ID: NCT03195192) aimed at assessing the association between phosphoprotein-based kinase activity and response to first-line treatment with an FDA-approved CDK4/6 inhibitor in combination with ET in HR+/HER2− in MBC patients. Patients were enrolled at six US institutions including five academic centers and one community-based cancer hospital. The study was conducted according to the guidelines of the Declaration of Helsinki and approved by the Institutional Review Board. The study protocol was approved by the Chesapeake Institutional Review Board as a central IRB (Pro00017810) and by the IRBs of the participating sites (University of Alabama at Birmingham, Cedar-Sinai Medical Center, Sylvester Comprehensive Cancer Center, Abramson Cancer Center, Sidney Kimmel Cancer Center at Jefferson Health, Women& Infants Hospital of Rhode Island, Virginia Cancer Specialists, and University of Washington). All patients provided voluntary written informed consent before entering the study. Patients with histologically or cytologically documented HR+/HER2− breast adenocarcinoma according to ASCO/CAP guidelines and with evidence of loco-regional recurrence or metastatic disease were enrolled in the study. Patients were eligible to participate in the study if they were candidates to receive ET in combination with a CDK4/6 inhibitor as first-line treatment for their advanced disease. Patients were also considered eligible for study enrollment if they had already started treatment with a standard dose and schedule of a CDK4/6 inhibitor and ET and: (A) had not received a CDK4/6 inhibitor for more than 10 weeks prior to study enrollment, (B) had sufficient tissue to perform the proposed molecular analysis, and (C) met all other eligibility criteria (See Supplementary Table 1). Based on the study protocol, ET could be initiated up to 4 weeks prior to the initiation of treatment with a CDK4/6 inhibitor. Archive tumor tissue (obtained from a biopsy or surgical resection of a metastatic lesion done within 4 months from study enrollment) availability was required for patient participation in the study. A breast and/or axillary node biopsy was acceptable in patients presenting with de novo metastatic breast cancer if they have not had a soft tissue biopsy of a metastatic lesion.

The response was assessed every 12 (±2) weeks for the first 12 months of treatment using RECIST 1.1 criteria^63^. Patients who received two or more cycles (≥8 weeks) of ET in combination with a CDK4/6 inhibitor were considered evaluable for response assessment per study protocol.

The study was purposefully conceived to identify markers of rapid/short-term progression to treatment as this group of patients has the greatest need to be promptly directed to more effective therapeutic options. Thus, our trial protocol was specifically designed with a follow-up duration of 12 months. If disease progression did not occur within the first 12 months of treatment, patients continued to receive ET in combination with a CDK4/6 inhibitor off protocol as per standard of care.

At the time of the study, three CDK4/6 inhibitors were approved by the FDA, namely palbociclib, ribociclib, and abemaciclib. For each patient, a CDK4/6 inhibitor was initiated in combination with ET using one of the following regimens: (A) palbociclib 125 mg, orally once daily on day 1 to 21 of every 28-day cycle followed by 7 days off treatment; (B) ribociclib 600 mg orally once daily on day 1 to 21 of every 28-day cycle followed by 7 days off treatment; and (C) abemaciclib 150 mg orally twice daily on day 1 to 28 of every 28-day cycle. Aromatase inhibitors were administrated orally and daily at a dose of 2.5 mg for letrozole, 1 mg for anastrozole, and 25 mg for exemestane. Fulvestrant, 500 mg, was administered intramuscularly on days 1, and 15 of cycle 1, and on day 1 of all subsequent 28-day cycles.

Biomarker analyses were conducted on pre-treatment formalin-fixed, paraffin-embedded (FFPE) diagnostic tissue samples collected within 4 months prior to study enrollment. For patients diagnosed with de novo metastatic disease, a biopsy of a breast and/or axillary node was considered acceptable for molecular analysis. Cancer cells and surrounding stroma/immune cells were isolated using laser capture microdissection (LCM) and downstream phosphoprotein-based kinase activity was measured using the reverse phase protein microarray (RPPA). To identify signaling events associated with response to treatment, functional phosphoprotein-based measurements of kinases and downstream substrates were correlated with 1-year progression-free survival (PFS). At the time of progression, patients were eligible for an optional biopsy of the metastatic lesion used for molecular profiling including targeted exome sequencing performed by a commercial CAP/CLIA laboratory (Caris Life Science) and RPPA-based functional mapping. Molecular profiles collected at the time of recurrence were used to inform future treatment selections.

The primary objective of the study was to evaluate whether baseline levels of phosphorylated Rb at the (S780) residue was associated with response to first-line treatment with palbociclib, ribociclib, or abemaciclib in combination with ET in ER+/HER2− MBC patients. The secondary objective of the study was to assess whether baseline expression or activation levels of seven substrates and regulators of CDK4/6 activity were associated with response to treatment. This preselected biomarker panel included: unmodified Rb, cyclin D1, p16INK, p27KIP, and post-translationally modified cyclin D1 (S286), p27KIP (T187), and FoxM1 (T600). As per the study protocol, an exploratory analysis was also conducted to assess the association between response to treatment and kinase-driven signaling events known to be involved in carcinogenesis and metastatic progression.

### Laboratory assays

FFPE blocks were provided to the testing laboratory by the enrolling institutions and freshly cut 8 µm sections were prepared from each tissue block. Tissue sections were mounted on uncharged glass slides and microdissected within a week. Representative slides were stained with Hematoxylin (Sigma Aldrich, St. Louis, MO) and Eosin (Sigma Aldrich, St. Louis, MO) for histopathological evaluation by a certified pathologist (LL).

Immediately before dissection, FFPE sections were deparaffinized in xylene (Sigma Aldrich, St. Louis, MO) for 30 min and hydrated in serial dilutions of ethanol (100, 95, and 70%) (Sigma Aldrich, St. Louis, MO)^64^. Samples were then rinsed in deionized water, stained with Hematoxylin (Sigma Aldrich, St. Louis, MO) and Scotts’ tap water (Electron Microscopy Sciences, Hatfield, PA), dehydrated in ethanol (70, 95, and 100%) and xylene, and dried at room temperature, as previously described^52,65^. A complete mini protease inhibitor cocktail (Roche Applied Science, Indianapolis, IN) was added to the 70% ethanol, water, hematoxylin, and Scott’s tap water substitute to preserve the phosphoproteome.

Cancer cells and surrounding stroma/immune cells were isolated under direct visualization using the PixCell II System (Arcturus, Mountain View, CA, USA) equipped with an infrared laser. Microdissected cells were captured on CapSure Macro LCM Caps (Applied Biosystems, Foster City, CA) and tumor epithelia and surrounding stroma/immune cells were captured on separate LCM Caps and processed as separate entities. We have previously demonstrated that the LCM protocol described above does not affect protein expression or activation and can be used for downstream phosphoproteomic analysis^66,67^.

Microdissected samples were lysed using the commercially available QProteome kit (Qiagen, Hilden, Germany). Approximately 1 µl of buffer was added to every ~250 captured cells. Following the manufacturer’s instructions, samples were incubated at 4 °C for 5 min and boiled for 20 min in a heating block. Cell lysates were placed in an 80 °C water bath for 2 h followed by 1 min incubation on ice. Samples were then centrifuged at 14,000 × *g* for 15 min at 4 °C and supernatants were collected and stored at −80 °C and batch analyzed at the completion of the study^52^.

At trial completion, samples were immobilized onto nitrocellulose-coated glass slides (Grace Biolabs, Bend, OR) using the 2470 Aushon Arrayer (Quanterix, Billerica, MA) equipped with 185 µm pins. Each sample was immobilized in technical replicates (*n* = 3) along with reference standards and internal controls, as previously described^48,65^. To assess the protein concentration in each sample and for normalization purposes, selected arrays were stained using a Sypro Ruby Protein Blot Stain (Invitrogen, Waltham, MA), according to the manufacturer’s instruction^65^. The protein concentration of each sample was estimated against a four-point bovine serum albumin serial dilution curve with a starting concentration of 1 µg/µl printed along with the experimental samples.

Before immunostaining, slides were incubated with Reblot Plus Mild Antibody stripping solution (MilliporeSigma, Burlington, MA) for 15 min at room temperature, washed with PBS, and incubated in I-block solution (Applied Biosystems, Foster City, CA) for at least 4 h. The remaining steps were performed on an automated system (Dako Cytomation, Carpinteria, CA) and included: (A) blocking of endogenous proteins that may interfere with the detection system, (B) antibody staining, (C) signal amplification using a tyramide-based commercially available kit (Dako GenPoint, Agilent, Santa Clara, CA,)^65^, and (D) fluorescent detection, as previously described^65^.

After endogenous proteins were blocked, each array was probed with one primary antibody recognizing an unmodified and post-translationally modified epitope of a target protein. A total of 126 primary antibodies were used to measure qualifying and exploratory biomarkers (Supplementary Table 2). Antibody specificity against the target epitopes was rigorously assessed as previously described^68^. After incubation with the primary antibody, arrays were probed with a biotinylated goat anti-rabbit (Vector Laboratories; 1:7500) or rabbit anti-mouse (Vector Laboratories; 1:7500) secondary antibody matching the species of the primary antibody. Selected arrays were probed with the secondary antibody only to account for background and unspecific binding of the staining reagents. A commercially available tyramine-based amplification system coupled with a streptavidin-conjugated IRDye680 fluorescence dye (LI-COR Biosciences, Lincoln, NE) was then used for the detection of the signal. Antibody- and Sypro Ruby Protein Blot-stained arrays were scanned using a laser scanner (TECAN, Männedorf, Switzerland) and images were analyzed with commercially available software (MicroVigene v5.1.0.0, VigeneTech, Inc.)^65,68^. The software automatically performs spot finding, subtraction of local background and unspecific signal, normalization to the amount of protein detected in the Sypro Ruby Protein Blot-stained arrays, and average across replicates.

### Outcome analysis and statistical considerations

Expression and/or activation of qualifying and exploratory biomarkers was compared between responders, which included patients with complete response (CR), partial response (PR), and stable disease (SD), and non-responders or patients that experience progressive disease (PD) within 12 months from treatment initiation. Biomarker levels were dichotomized as above/below the population median. The chi-square test was used to compare the prevalence of high/low expression/activation of each biomarker in responders and non-responders. Continuous RPPA relative intensity values were displayed using box plots and scatter plots generated in GraphPad Prism v9.4; *p* values of two-sided chi-square tests <0.05 were considered significant. Continuous RPPA values were also displayed using unsupervised hierarchical clustering analysis generated in JMP v16 (SAS Institute Inc.) where data were normalized according to Ward’s method.

### Reporting summary

Further information on research design is available in the Nature Research Reporting Summary linked to this article.

## Supplementary information

Supplementary Material

REPORTING SUMMARY

## Data Availability

The RPPA datasets generated by the current study are available from the corresponding author and can be accessed on the Side-Out Foundation Bioinformatics Portal at https://sideoutfoundation.gmu.edu.

## Code availability

Custom code or new mathematical algorithms were not used for this analysis.
