# Supplementary material for: AKT/mTOR signaling modulates resistance to endocrine therapy and CDK4/6 inhibition in metastatic breast cancers
Source: NPJ Precis Oncol. 2023 Feb 16;7:18. doi: 10.1038/s41698-023-00360-5 (PMC9935518; doi:10.1038/s41698-023-00360-5)
Supplement: Supplementary file 1 — Supplementary Material [file 41698_2023_360_MOESM1_ESM.pdf]

SUPPLEMENTARY MATERIAL

Supplementary Figure 1. Expression and activation levels of selected cell cycle regulators based on patients' outcomes. Box plots with median (center line) and maximum and minimum values (whiskers) show increased expression and activation levels of proliferation markers in patients with progressive disease compared to responders (Panel A). Similar results were also detected for phosphorylation levels of the S118 residue of the Estrogen Receptor alpha (Panel B). Samples are color-coded based on patient's outcome.

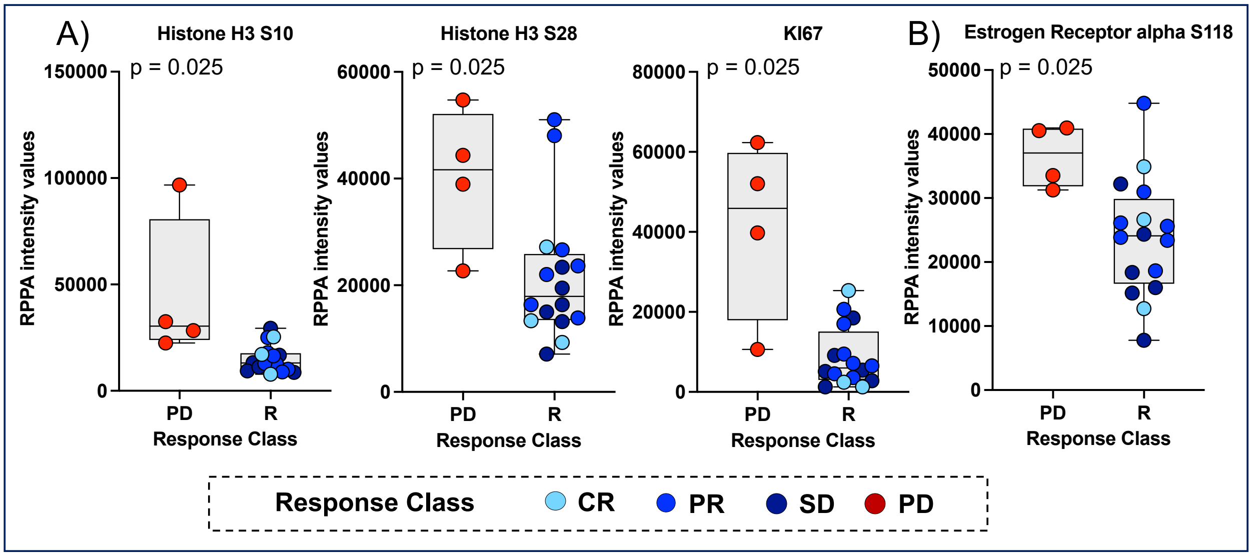

Supplementary Figure 2. Unsupervised hierarchical clustering analysis of unmodified and post-translationally modified kinases and their substrates in the tumor epithelia of MBC patients based on site of biopsy. RPPA relative intensity values are displayed on a scale ranging from red to blue, where red indicates high levels and blue indicates low levels of expression/activation. On the x-axis are listed proteins measured by RPPA; on the y-axis patients' outcome were color-coded based on response where blue indicates responders (CR, PR, and SD) and red non-responders (PD).

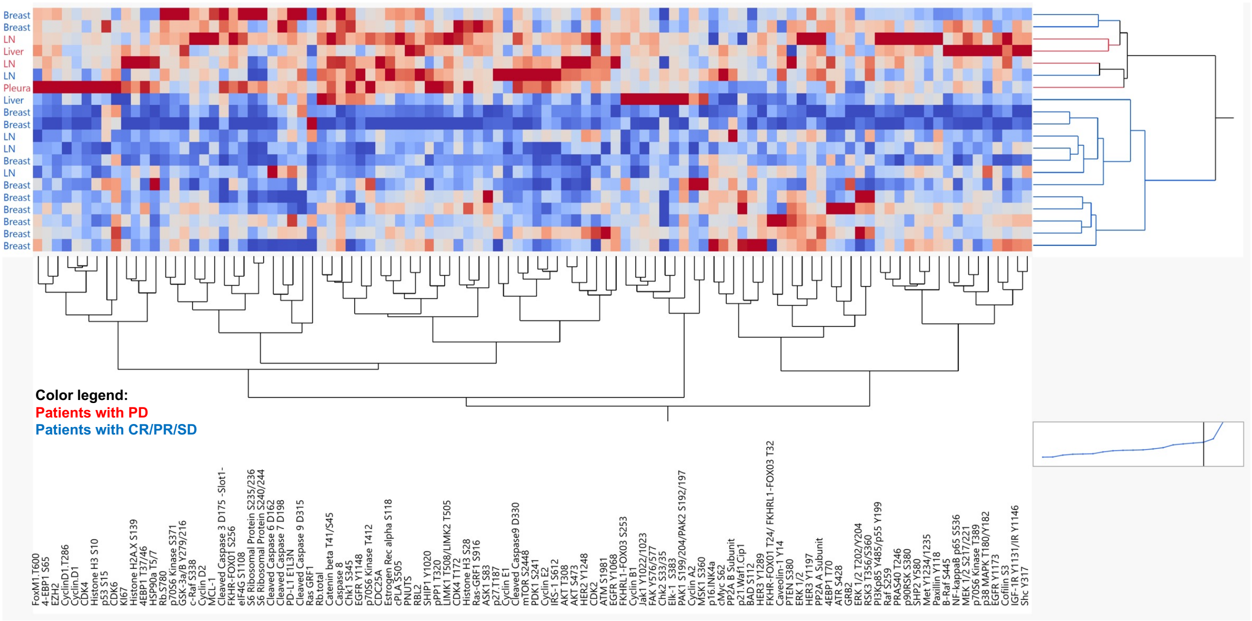

Supplementary Figure 3. Example of the standardized format of the RPPA assay. Absolute RPPA intensity values for the experimental samples analyzed in this trial are reported as relative intensity units of a reference standard curve immobilized along with the experimental samples. Cut-off values can be established based on the sample distribution and outcome and can be directly tested in independent cohorts of samples

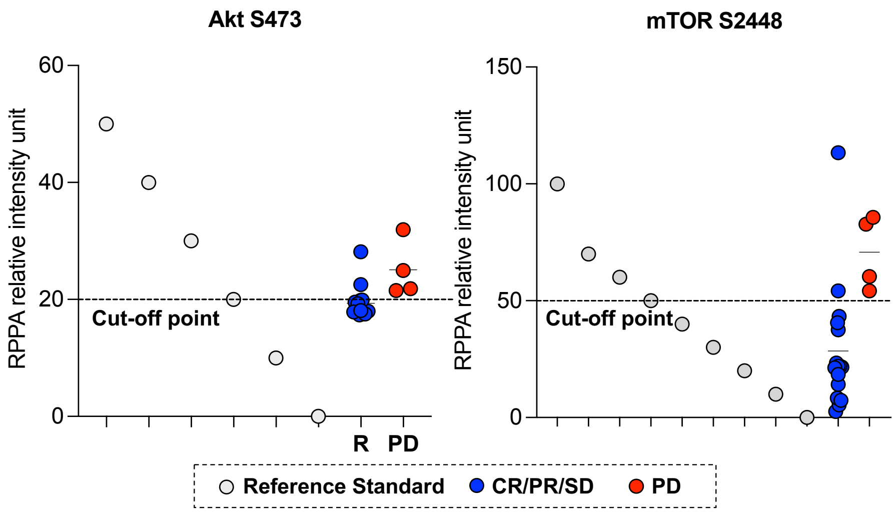

Supplementary Table 1. List of inclusion and exclusion criteria used to determine patients' eligibly for the study.

Inclusion criteria
Have a histologically or cytologically proven diagnosis of HR+/HER2- breast adenocarcinoma according to ASCO/CAP guidelines.
Have evidence of loco-regional recurrence or metastatic disease.
Be candidates to receive endocrine therapy and palbociclib, ribociclib or abemaciclib as first-line treatment for their loco-regionally recurrent or metastatic breast cancer and have not received this treatment for longer than 4 weeks from time of study enrollment.
Have measurable disease by RECIST v.1.1. For patients with bone disease only, bone lesions must be confirmed by CT, MRI, or bone X-ray.
Have no prior treatment with chemotherapy for loco-regionally recurrent or metastatic breast cancer.
Be ≥ 18 years of age.
Have a ECOG score of 0-1.
Be postmenopausal or receiving medical ovarian suppression with luteinizing hormone-releasing hormone (LHRH) agonists.
Have archive tumor tissue obtained from a biopsy or surgical resection of a metastatic lesion done within 4 months from study enrollment. A breast and/or axillary node biopsy is acceptable in patients presenting with de novo metastatic disease.
Understand and provide written informed consent prior to initiation of any study-specific procedures.
**Exclusion criteria**
Lack of archive tumor tissue from a biopsy or surgical resection of a metastatic lesion done within 4 months of study enrollment. Patients will be given an option to have a repeated biopsy of a metastatic lesion.
Have symptomatic CNS metastasis. Patients with a history of CNS metastases who have been treated with whole brain irradiation must be stable without symptoms for 4 weeks after completion of treatment, with image documentation required, and must be either off steroids or on a stable dose of steroids for ≥ 4 weeks prior to enrolment.
Have uncontrolled concurrent illness including, but not limited to, ongoing or active serious infection, symptomatic congestive heart failure, unstable angina pectoris, unstable cardiac arrhythmias, psychiatric illness, or situations that would limit compliance with the study requirements or ability to willingly give written informed consent.

Supplementary Table 2. List of antibodies, along with vendor, species, and p values for the 126 analytes included in the analysis.

Biomarker	N. of cases	P value	Vendor	Cat #	Dilution	Host
4EBP1 S65	20	***0.025***	Cell Signaling	9451	1:200	R
4EBP1 T37/46	20	***0.025***	Cell Signaling	9459	1:500	R
4EBP1 T70	20	1.000	Cell Signaling	9455	1:200	R
AKT S473	20	***0.025***	Cell Signaling	9271	1:100	R
AKT T308	20	***0.025***	Cell Signaling	9275	1:100	R
ASK1 S83	20	0.264	Cell Signaling	3761	1:50	R
ATM S1981	20	0.264	Cell Signaling	5883	1:50	RmAb
ATR S428	20	***0.025***	Cell Signaling	2853	1:50	R
B-Raf S445	20	***0.025***	Cell Signaling	2696	1:50	R
BAD S112	20	0.264	Cell Signaling	9291	1:200	R
c-Raf S338	20	0.264	Cell Signaling	9427	1:200	RmAb
Caspase 8	20	0.264	Cell Signaling	9746	1:1000	M
Catenin beta T41/S45	20	***0.025***	Cell Signaling	9565	1:50	R
Caveolin 1 Y14	20	1.000	Epitomics	2267-1	1:200	RmAb
CDC25A S76	20	***0.025***	Sigma	SAB4300114	1:100	R
CDK2	20	***0.025***	Cell Signaling	2546	1:200	R
CDK4	20	***0.025***	Cell Signaling	12790	1:200	RmAb
CDK4 T172	20	***0.025***	Biosource	MBS9126724	1:2000	R
CDK6	20	1.000	Cell Signaling	13331	1:50	RmAb
Chk1 S345	20	***0.025***	Cell Signaling	2341	1:50	R
Chk2 S33/35	20	0.264	Cell Signaling	2665	1:50	R
Cleaved Caspase 3 D175	20	***0.025***	Cell Signaling	9661	1:50	R
Cleaved Caspase 6 D162	20	0.264	Cell Signaling	9761	1:50	R
Cleaved Caspase 7 D198	20	0.264	Cell Signaling	9491	1:100	R
Cleaved Caspase 9 D315	20	***0.025***	Cell Signaling	9505	1:100	R
Cleaved Caspase 9 D330	20	***0.013***	Cell Signaling	9501	1:50	R
c-Myc S62	20	0.264	Cell Signaling	13748	1:100	RmAb
Cofilin S3	20	0.264	Cell Signaling	3313	1:500	RmAb
cPLA S505	20	***0.025***	Cell Signaling	2831	1:1000	R
Cyclin A2	20	0.264	DAKO	4656	1:50	M
Cyclin B1	20	***0.025***	Cell Signaling	4135	1:200	M
Cyclin D1	20	0.822	BD	554180	1:100	M
Cyclin D2	20	1.000	Cell Signaling	3741	1:100	R
Cyclin E1	20	***0.025***	Cell Signaling	20808	1:100	R
Cyclin E2	20	***0.025***	Cell Signaling	4132	1:200	R

CyclinD1 T286	20	***0.025***	Cell Signaling	3300	1:500	R
EGFR Y1068	20	***0.025***	Cell Signaling	2234	1:50	R
EGFR Y1148	20	0.264	Biosource	44-792	1:100	R
EGFR Y1173	20	***0.025***	Biosource	44-794	1:100	R
eIF4G S1108	20	***0.025***	Cell Signaling	2441	1:1000	R
Elk-1 S383	20	***0.025***	Cell Signaling	9181	1:100	R
ERK 1/2	20	0.264	Cell Signaling	9102	1:200	R
ERK 1/2 T202/Y204	20	0.264	Cell Signaling	9101	1:1000	R
Estrogen Rec alpha S118	20	***0.025***	Cell Signaling	2511	1:1000	M
EZH2	20	***0.025***	Cell Signaling	5246	1:500	R
FAK Y576/577	20	***0.025***	Cell Signaling	3281	1:200	R
FOX01 S256	20	***0.025***	Cell Signaling	9461	1:100	R
FOX01 T24/FOX03 T32	20	***0.025***	Cell Signaling	9464	1:200	R
FOX03 S253	20	***0.025***	Upstate	06-953	1:1000	R
FoxM1 T600	20	***0.025***	Cell Signaling	14655	1:100	RmAb
GRB2	20	1.000	Cell Signaling	3972	1:1000	R
GSK 3a/B Y279/216	20	0.264	BioSource	44-604	1:500	R
HER2 Y1248	20	0.264	Imgenex	IMG-90189	1:500	R
HER3 Y1197 (C56E4)	20	0.264	Cell Signaling	4561	1:100	RmAb
HER3 Y1289 (21D3)	20	1.000	Cell Signaling	4791	1:200	RmAb
Histone H2A.X S139	20	***0.025***	Cell Signaling	9718	1:50	R
Histone H3 Acetyl K14	17	***0.043***	Millipore	07-353	1:10000	R
Histone H3 Acetyl K8	17	***0.043***	Cell Signaling	2594	1:200	R
Histone H3 Acetyl K9/14	17	0.453	Cell Signaling	9677	1:2000	R
Histone H3 Di-Methyl K9	17	0.453	Cell Signaling	9753	1:500	R
Histone H3 Pan-Methyl K9	17	***0.043***	Cell Signaling	4069	1:100	R
Histone H3 S10	20	***0.025***	Upstate	06-570	1:200	R
Histone H3 S28	20	***0.025***	Upstate	07-145	1:1000	R
Histone H3 Tri Methyl K27	17	***0.043***	Millipore	07-449	1:2500	R
HLA-DR	17	0.072	Santa Cruz	sc-53319	1:50	M
HLA-DR/DP/DQ/DX	17	0.072	Santa Cruz	sc-53302	1:50	M
HSP90a T5/7	20	***0.025***	Cell Signaling	3488	1:100	R
IGF-1R Y1131/IR Y1146	20	0.264	Cell Signaling	3021	1:500	R
IRS-1 S612	20	***0.025***	Cell Signaling	2386	1:200	R
Jak1 Y1022/1023	20	0.264	Cell Signaling	3331	1:50	R
Jak2 Y1007	17	0.453	Cell Signaling	4406	1:200	RmAb
Ki67	20	***0.025***	DAKO	M7240	1:100	M
LIMK1 T508/LIMK2 T505	20	***0.025***	Cell Signaling	3841	1:100	R
MCL-1	20	0.264	Cell Signaling	5453	1:200	R

MEK 1/2 S217/221	20	***0.025***	Cell Signaling	9121	1:200	R
Met Y1234/1235	20	0.264	Cell Signaling	3126	1:200	R
MHC class I	17	0.600	Santa Cruz	SC-55582	1:500	M
MSK1 S360	20	1.000	Cell Signaling	9594	1:50	R
mTOR S2448	20	***0.025***	Cell Signaling	2971	1:100	R
NF-kappaB (p65) S536	20	***0.025***	Cell Signaling	3033	1:200	RmAb
Notch1	17	***0.043***	Millipore	07-1231	1:500	R
p16 INK4a	20	0.264	Millipore	MABE1328	1:50	M
p21 Waf1/Cip1	20	1.000	Cell Signaling	2946	1:200	M
p27 Kip1	17	0.453	BD	610242	1:100	M
p27 T187	20	0.264	Zymed	71-7700	1:200	R
p38 MAPK T180/Y182	20	0.264	Cell Signaling	9211	1:100	R
p53 S15	20	0.264	Cell Signaling	9284	1:1000	R
p70S6 Kinase S371	20	0.264	Cell Signaling	9208	1:50	R
p70S6 Kinase T389	20	***0.025***	Cell Signaling	9205	1:100	R
p70S6 Kinase T412	20	0.264	Upstate	07-018	1:500	R
p90RSK S380	20	***0.025***	Cell Signaling	9341	1:200	R
PAK1 S199/204 PAK2 S192/197	20	0.264	Cell Signaling	2605	1:50	R
Paxillin Y118	20	0.264	Cell Signaling	2541	1:500	R
PD-L1	20	***0.025***	Cell Signaling	13684	1:500	R
PDK1 S241	20	***0.025***	Cell Signaling	3601	1:200	R
PI3Kp85 Y485/p55 Y199	20	***0.025***	Cell Signaling	4228	1:100	R
PLCgamma1 Y783	18	0.257	Cell Signaling	2821	1:100	R
PLK1 T210	17	0.323	BD	558400	1:200	M
PNUTS	20	***0.025***	Cell Signaling	14171	1:200	R
PP2A A Subunit	20	0.264	Cell Signaling	2039	1:1000	R
PP2A B Subunit	20	0.264	Cell Signaling	4953	1:1000	R
p-PP1 T320	20	***0.025***	Cell Signaling	2581	1:100	R
PRAS40 T246	20	***0.013***	Biosource	44-1100	1:1000	R
PTEN S380	20	1.000	Cell Signaling	9551	1:500	R
Raf S259	20	***0.025***	Cell Signaling	9421	1:100	R
Ras-GRF1	20	0.264	Cell Signaling	3322	1:200	R
Ras-GRF1 S916	20	***0.025***	Cell Signaling	3321	1:50	R
Rb S780	20	***0.025***	Cell Signaling	3590	1:2000	R
Rb	20	***0.025***	Cell Signaling	9309	1:500	M
RBL2	20	0.264	Cell Signaling	13610	1:100	R
RSK3 T356/S360	20	1.000	Cell Signaling	9348	1:500	R
S6 Ribosomal Protein S235/236	20	0.264	Cell Signaling	4856	1:200	R
S6 Ribosomal Protein S240/244	20	0.264	Cell Signaling	2215	1:1000	R

SAPK/JNK T183/Y185	19	***0.018***	Cell Signaling	9251	1:100	R
SEK1/MKK4 S80	19	0.906	Cell Signaling	9155	1:50	R
Shc Y317	20	0.264	Upstae	07-206	1:200	R
SHIP1 Y1020	20	0.264	Cell Signaling	3941	1:50	R
SHP2 Y580	20	0.264	Biosource	44-558	1:500	R
Src Family Y416	18	0.257	Cell Signaling	2101	1:100	R
Src Y527	18	***0.023***	Cell Signaling	2105	1:200	R
Stat1 Y701	18	0.257	Cell Signaling	9171	1:500	R
Stat3 Y705	17	0.453	Cell Signaling	9145	1:100	RmAb
Stat3 Y727	17	0.072	Cell Signaling	9134	1:100	R
Stat5 Y694	18	0.257	Cell Signaling	9351	1:50	R
Stat6 Y641	18	0.163	Cell Signaling	9361	1:100	R
YAP S127	17	0.453	Cell Signaling	13008	1:100	RmAb

Supplementary Table 3. List of adverse events experienced by the trial participants.

Adverse Event (AE)	CTCAE Grade	Due to study protocol	Number of AE
Bilateral oophorectomy	NA	Not related	1
Cough	Grade 2	Not related	1
Creatinine increased	Grade 2/3	Not related	1
Cyberknife Radiation	NA	Not related	1
Dyspnea	Grade 2	Not related	1
Dyspnea on exertion	Grade 1	Not related	1
Flu-like symptoms	Grade 2	Not related	1
Hip pain	Grade 2	Not related	1
Kyphoplasty	NA	Not related	1
Lung Infection	Grade 2	Not related	1
UTI	Grade 2	Not related	1
Confusion	Grade 2	Unlikely related	1
UTI	Grade 2	Unlikely related	1
Dyspnea	Grade 2	Possibly related	1
Sinusitis	Grade 2	Possibly related	1
Nausea	Grade 2	Probably related	1
Vomiting	Grade 2	Probably related	1
Neutropenia	Grade 3	Definitely related	5
WBC decreased and neutropenia	Grade 3	Definitely related	1
