# Supplementary material for: AKT/mTOR signaling modulates resistance to endocrine therapy and CDK4/6 inhibition in metastatic breast cancers
Source: NPJ Precis Oncol. 2023 Feb 16;7:18. doi: 10.1038/s41698-023-00360-5 (PMC9935518; doi:10.1038/s41698-023-00360-5)
Supplement: Supplementary file 2 — REPORTING SUMMARY [file 41698_2023_360_MOESM2_ESM.pdf]

nature portfolio

Corresponding author(s): Mariaelena Pieroon

Last updated by author(s): 2023/01/06

Reporting Summary

Nature Portfolio wishes to improve the reproducibility of the work that we publish. This form provides structure for consistency and transparency in reporting. For further information on Nature Portfolio policies, see our Editorial Policies and the Editorial Policy Checklist.

Statistics

For all statistical analyses, confirm that the following items are present in the figure legend, table legend, main text, or Methods section.

n/a	Confirmed	
☐	☒	The exact sample size (*n*) for each experimental group/condition, given as a discrete number and unit of measurement
☐	☒	A statement on whether measurements were taken from distinct samples or whether the same sample was measured repeatedly
☐	☒	The statistical test(s) used AND whether they are one- or two-sided<br>*Only common tests should be described solely by name; describe more complex techniques in the Methods section.*
☐	☒	A description of all covariates tested
☐	☒	A description of any assumptions or corrections, such as tests of normality and adjustment for multiple comparisons
☐	☒	A full description of the statistical parameters including central tendency (e.g. means) or other basic estimates (e.g. regression coefficient) AND variation (e.g. standard deviation) or associated estimates of uncertainty (e.g. confidence intervals)
☐	☒	For null hypothesis testing, the test statistic (e.g. *F*, *t*, *r*) with confidence intervals, effect sizes, degrees of freedom and *P* value noted<br>*Give P values as exact values whenever suitable.*
☒	☐	For Bayesian analysis, information on the choice of priors and Markov chain Monte Carlo settings
☒	☐	For hierarchical and complex designs, identification of the appropriate level for tests and full reporting of outcomes
☒	☐	Estimates of effect sizes (e.g. Cohen's *d*, Pearson's *r*), indicating how they were calculated

Our web collection on statistics for biologists contains articles on many of the points above.

Software and code

Policy information about availability of computer code

Data collection	No software was used
Data analysis	GraphPad Prism v9.4<br>JMP v16 (SAS Institute)

For manuscripts utilizing custom algorithms or software that are central to the research but not yet described in published literature, software must be made available to editors and reviewers. We strongly encourage code deposition in a community repository (e.g. GitHub). See the Nature Portfolio guidelines for submitting code & software for further information.

Data

Policy information about availability of data

All manuscripts must include a data availability statement. This statement should provide the following information, where applicable:

- Accession codes, unique identifiers, or web links for publicly available datasets
- A description of any restrictions on data availability
- For clinical datasets or third party data, please ensure that the statement adheres to our policy

The datasets generated by the current study are available from the corresponding author on reasonable request.

Human research participants

Policy information about studies involving human research participants and Sex and Gender in Research.

Reporting on sex and gender	All 20 evaluable metastatic breast cancer patients were female and the median age was 67.5 (range between 36-79).
Population characteristics	We have provided the following patients characteristics: age, sex, race, target lesion, tumor histology, site of metastasis, and treatment received as part of Table 1 of the manuscript.
Recruitment	Patients with hormone receptor metastatic breast cancer were screened and offered study participation by the treating physician and research team at each of the participating sites. We are not aware of any selection bias.
Ethics oversight	The study protocol was approved by a central IRB and then by local IRB at each of the participating sites (University of Alabama at Birmigham, Cedars-Sinai Medical Center, Sylvester Comprehensive Cancer Center, Sidney Kimmel Cancer Center at Thomas Jefferson University, Abramson Cancer Center at University of Pennsylvania, Women and Infants Hospital of Rhode Island, Virginia Cancer Specxialists, ans University of Washington .

Note that full information on the approval of the study protocol must also be provided in the manuscript.

Field-specific reporting

Please select the one below that is the best fit for your research. If you are not sure, read the appropriate sections before making your selection.

☒ Life sciences ☐ Behavioural & social sciences ☐ Ecological, evolutionary & environmental sciences

For a reference copy of the document with all sections, see nature.com/documents/nr-reporting-summary-flat.pdf

Life sciences study design

All studies must disclose on these points even when the disclosure is negative.

Sample size	While the study was originally designed to enroll 100 patients to achieve a statistical power of 80% for the qualifying biomarker analysis, due to slow accrual, the study was prematurely closed.
Data exclusions	Of the 41 patients screened, 27 met study eligibility criteria and 20 of 27 patients were evaluable for response assessment as outlined per study protocol and were included in this biomarker analysis. Of the 7 patients that were not included in the final analysis, two had insufficient biological material, two withdrew from the study, two became eligible for surgery and one had a change of the treatment plan.<br>For the RPPA analysis, a few samples were insufficient to complete the full analysis and were not included in a limited number of comparisons.
Replication	RPPA data were generated in technical replicates and average values are reported in the manuscript. CV across technical replicates were evaluated for each patient and analyte.
Randomization	Not Applicable
Blinding	Not Applicable

Reporting for specific materials, systems and methods

We require information from authors about some types of materials, experimental systems and methods used in many studies. Here, indicate whether each material, system or method listed is relevant to your study. If you are not sure if a list item applies to your research, read the appropriate section before selecting a response.

Materials & experimental systems

n/a	Involved in the study	
☐	☒	Antibodies
☒	☐	Eukaryotic cell lines
☒	☐	Palaeontology and archaeology
☒	☐	Animals and other organisms
☐	☒	Clinical data
☒	☐	Dual use research of concern

Methods

n/a	Involved in the study	
☒	☐	ChIP-seq
☒	☐	Flow cytometry
☒	☐	MRI-based neuroimaging

Antibodies

Antibodies used	Supplementary Table 2 lists of antibodies, along with vendor, catalogue number, dilution used, and species for the 126 analytes measured by RPPA
Validation	Antibody specificity against the target epitopes was tested on a panel of cell lines and/or human samples using conventional Western blotting technique and tested on the arrays to assure the linear dynamic range of the analytes was captured and the signal:noise ratio was within an acceptable range.

Clinical data

Policy information about clinical studies

All manuscripts should comply with the ICMJE guidelines for publication of clinical research and a completed CONSORT checklist must be included with all submissions.

Clinical trial registration	Clinical trial.gov ID: NCT03195192
Study protocol	The study synopsis and eligibility criteria are found on Clinical Trials.gov. The full protocl is available at each of the partcipating institutions and can be provided for review on request.
Data collection	Study start date 2017-03-09, study completion 2020-06-03. Clinica data was collected at each of the participating sites and entered into a central database at the Sidney Kimmel Cancer Center at Thomas Jefferson University. Tissue was collected at participating sites and sent to Goerge Mason University for analysis.
Outcomes	Response was assessed every 12 (± 2) weeks for the first 12 months of treatment using RECIST 1.1 criteria. Patients who received two or more cycles (>8 weeks) of ET in combination with a CDK4/6 inhibitor were considered evaluable for response assessment per study protocol. Baseline biopsies were used to assess distribution of qualifying and exploratory biomarkers in responders and non-responders.
